# *De Novo* Genome Sequence Assembly of Dwarf Coconut (*Cocos nucifera* L. ‘Catigan Green Dwarf’) Provides Insights into Genomic Variation Between Coconut Types and Related Palm Species

**DOI:** 10.1534/g3.119.400215

**Published:** 2019-06-05

**Authors:** Darlon V. Lantican, Susan R. Strickler, Alma O. Canama, Roanne R. Gardoce, Lukas A. Mueller, Hayde F. Galvez

**Affiliations:** * Genetics Laboratory, Institute of Plant Breeding, College of Agriculture and Food Science, University of the Philippines Los Baños, College, Laguna, Philippines 4031; † Philippine Genome Center, University of the Philippines System, Diliman, Quezon City, Philippines; ‡ Boyce Thompson Institute, Ithaca, New York 14853, and; § Institute of Crop Science, College of Agriculture and Food Science, University of the Philippines Los Baños, College, Laguna, Philippines 4031

**Keywords:** *Cocos nucifera* L., dwarf coconut, genome assembly, Illumina Miseq Sequencing, PacBio SMRT sequencing, Dovetail Chicago sequencing, hybrid assembly, SSR and SNP markers

## Abstract

We report the first whole genome sequence (WGS) assembly and annotation of a dwarf coconut variety, ‘Catigan Green Dwarf’ (CATD). The genome sequence was generated using the PacBio SMRT sequencing platform at 15X coverage of the expected genome size of 2.15 Gbp, which was corrected with assembled 50X Illumina paired-end MiSeq reads of the same genome. The draft genome was improved through Chicago sequencing to generate a scaffold assembly that results in a total genome size of 2.1 Gbp consisting of 7,998 scaffolds with N50 of 570,487 bp. The final assembly covers around 97.6% of the estimated genome size of coconut ‘CATD’ based on homozygous k-mer peak analysis. A total of 34,958 high-confidence gene models were predicted and functionally associated to various economically important traits, such as pest/disease resistance, drought tolerance, coconut oil biosynthesis, and putative transcription factors. The assembled genome was used to infer the evolutionary relationship within the palm family based on genomic variations and synteny of coding gene sequences. Data show that at least three (3) rounds of whole genome duplication occurred and are commonly shared by these members of the *Arecaceae* family. A total of 7,139 unique SSR markers were designed to be used as a resource in marker-based breeding. In addition, we discovered 58,503 variants in coconut by aligning the Hainan Tall (HAT) WGS reads to the non-repetitive regions of the assembled CATD genome. The gene markers and genome-wide SSR markers established here will facilitate the development of varieties with resilience to climate change, resistance to pests and diseases, and improved oil yield and quality.

Coconut, a diploid (2n = 32) crop, is the only recorded species under the genus *Cocos* which is a member of the family *Areca*ceae and sub-family Arecoideae (Nambiar and Swaminathan 1960; Abraham and Mathew 1963). It is a major agricultural crop in humid areas in the tropics, with recorded annual worldwide production of 59 million tons (FAOSTAT 2016). South East Asia accounts for 91% of the global agricultural area planted to coconut. The Philippines is the major exporter of coconut products, especially coconut oil, and the second largest producer next to Indonesia with a total production of 14.7 million metric tons or 25% of the world’s production (FAOSTAT 2016; PSA 2016).

Coconut is monospecific and consists of two ecotypes based on stature and breeding habit, the ‘Talls’ and ‘Dwarfs’ (Menon and Pandalai 1958). Generally, the ‘Talls’ are highly outcrossing while ‘Dwarfs’ are highly selfed; hybrids between these two display high heterosis (Perera *et al.* 2000). There are two main classes of ‘Tall’ – ‘Nie kafa’ and ‘Nie vai’. The former evolved naturally and was disseminated through ocean currents while the latter is a result of cultivation and dissemination by human activities (Harries 1981). The origin of ‘Dwarf’, on the other hand, is inconclusive, but it is typically found in areas near human habitats and has traits associated with human selection (Perera *et al.* 2000; Bourdeix *et al.* 2001). However, a recent molecular marker study has determined differences in the SSR allele frequency between ‘Dwarf’ and ‘Tall’ accessions. This result, in addition to ethnobotanical and geographic information, indicates that the ‘Dwarf’ originated from ‘Tall’ through typical domestication events that occurred in Southeast Asia. Diversification likely occurred through autogamy followed by a series of allele fixations in a random subset of ancestral ‘Talls’ (Perera *et al.* 2016). Under natural conditions, ‘intermediate’ types can occur as a result of the sporadic crossing of traditional populations of ‘Talls’ and ‘Dwarfs’. This ‘intermediate’ type is phenotypically distinct from the two types and can be fixed as ‘semi-Talls’ with reproductive traits similar to ‘Dwarf’ but with faster growth (Batugal *et al.* 2005).

True to its distinction as “the tree of life”, the coconut tree has no shortage of uses for its parts, from the leaves down to the roots, whether domestic, commercial or industrial in nature. The fruit itself is used in a wide variety of products and by-products. The liquid endosperm, or coconut water, from the young fruit is gaining attention as a high value energy drink. The endocarp is used as alternative fuel source in the form of charcoal. The mesocarp is a source of fiber or coir for industrial uses. Finally, the solid endosperm or ‘meat’ from the mature fruit can be processed to yield copra oil, the most economically important product of coconut (Batugal *et al.* 2005).

Medium chain fatty acids (MCFAs) and long-chain fatty acids comprise around 84% of the total composition of copra oil, of which lauric acid (C12) is the most predominant (Padolina *et al.* 1987; Laureles *et al.* 2002). This high proportion of saturated fatty acids has been previously reported to increase cholesterol synthesis in the body thus increasing the risk of cardiovascular diseases (Mensink *et al.* 2003). However, this has been scientifically debunked by several studies demonstrating that coconut oil is not hypercholesterolemic and atherogenic (Blackburn *et al.* 1987; Kintanar and Castro 1988; Dayrit *et al.* 1989; Müller *et al.* 2003a; Müller *et al.* 2003b; Orsavova *et al.* 2015). To develop varieties with an improved fatty acid profile, the coconut oil biosynthesis pathway must be elucidated. KEGG (Kyoto Encyclopedia of Genes and Genomes) analysis from suppression subtractive hybridization (SSH) data showed that 1-acyl-sn-glycerol-3-phosphate acyltransferase (LPAAT), phospholipase D, Acetyl-CoA carboxylase carboxyltransferase beta subunit, 3-hydroxyisobutyryl-CoA hydrolase-like and pyruvate dehydrogenase E1 β subunit were associated with fatty acid biosynthesis or metabolism in coconut (Liang *et al.* 2014). Although coconut oil is primarily composed of C12, breeding for elevated MCFAs should be focused on C8 and C10 since there is less variation on the C12 composition among the Philippine coconut accessions (Laureles *et al.* 2002).

Worldwide coconut production has been constrained by several biotic (pests and diseases) and abiotic (*e.g.*, drought) factors. Diseases such as viruses, viroids, fungi, and phytoplasma and insect pests such as coconut scale insect, red palm weevil and rhinoceros beetle have remained major threats in several coconut-producing countries (Salim and Mahindapala 1981; Rohde *et al.* 1990; Hanold and Randles 1991; Jacob and Bhumannavar 1991; Howard and Oropeza 1998; Fiaboe *et al.* 2012; Watson *et al.* 2015; Cortaga *et al*. 2019). Transcriptomic studies in coconut have focused on the identification of genes and deciphering the molecular mechanisms of host plant resistance to major diseases, while focus on insect resistance have been very limited (Cardeña *et al.* 2003; Fan *et al.* 2013, Huang *et al.* 2014, Nejat *et al.* 2015). Resistance (*R*) genes encode a particular set of proteins with cytoplasmic nucleotide-binding site and leucine-rich repeat (NBS-LRR) domains, which are responsible for plant resistance against a wide range of pathogens and insect pests (Hammond-Kosack and Jones 1997; Kaloshian 2004; Klingler *et al.* 2005). Much work has been done to study the evolution of R-genes in several crops, as well as the coping mechanisms of diseases during the course of evolution (Li *et al.* 2010; Bouktila *et al.* 2015; Gu *et al.* 2015; Khan *et al.* 2016; Zheng *et al.* 2016). In coconut, candidate NBS-type resistance genes have been characterized but the transcriptome profiles were limited to coconut embryo, endosperm, and leaf samples (Puch-Hau *et al.* 2016). Genome-wide analysis of R-genes and associated sequences in coconut is therefore important in order to obtain a complete picture of the crop’s potential inherent defense system, and characterize each phylogenetic relationship in reference with characterized/validated plant R-genes.

Throughout its entire productive lifespan, the coconut palm is frequently exposed to soil and atmospheric drought (Bai and Rajagopal 2000). Several accounts of the negative impact of drought to coconut productivity as well as in its growth and physiology have been reported (Repellin *et al.* 1997; Bai and Rajagopal 2000; Prado *et al.* 2001; Passos *et al.* 2009; Gomes *et al.* 2009; Gomes and Prado 2010). Accumulation of sugars, amino acids, alcohols and quaternary ammonium are some of the characterized mechanisms crops use to withstand environmental (abiotic) stress (Morgan 1984). A high level of proline was found in drought tolerant coconut genotypes (Voleti *et al.* 1990). Gomes *et al.* (2010) demonstrated that proline is more related to a protective rather than an osmoregulatory mode of water stress response. Advancements in the field of genomics such as microarray and RNA-seq technologies were able to elucidate the molecular mechanisms of drought in rice, sorghum and *Arabidopsis* (Seki *et al.* 2002; Dugas *et al.* 2011; Wang *et al.* 2011). In coconut, a genome-wide network of drought response-related genes has not yet been established.

The draft genome assembly of coconut facilitates the identification of genes responsible for several economically important traits such as insect and disease resistance, and plant traits associated with agro-ecological adaptation such as resistance to drought stress (Fan *et al.* 2013). The whole genome sequences of two species closely related to coconut, date palm and oil palm, are currently available (Al-Mssallem *et al.* 2013; Singh *et al.* 2013). The genome of ‘Hainan Tall’ (HAT) coconut variety was recently published (Xiao *et al.* 2017). There are reports of draft genome assemblies of dwarf coconut but none have been published to date. The genome size of coconut is estimated to be around 2.6 Gbp and repetitive sequences may be between 50–70% of the total genome size (Alsaihati 2014). A flow cytometry study revealed that the ‘Catigan Green Dwarf’ (‘CATD’) variety of coconut has a genome size of 2.72 Gbp (1C) (Gunn *et al.* 2015), which is 4 and 1.6 times higher than date palm and oil palm, respectively.

In this paper, we present the first whole genome sequence of a ‘Dwarf’ type coconut represented by ‘Catigan Green Dwarf’ (CATD) coconut variety, chosen for its genome simplicity and low heterozygozity. PacBio Single Molecule, Real-Time (SMRT) sequencing was generated at 15X coverage of the estimated genome size and corrected with assembled 50X Illumina paired-end MiSeq reads. Scaffolds were generated through an *in vitro* proximity ligation method. We aimed to characterize the gene units in coconut and maximize the utility of this genome information for gene discovery, molecular marker development, and routine marker-assisted breeding applications.

## Materials and Methods

### Preparation of DNA material and outsourced sequencing

Total genomic DNA samples of ‘Catigan Green Dwarf’ (CATD) coconut variety (Palm number 1817) from Philippine Coconut Authority (PCA) - Zamboanga Research Center (ZRC), Philippines were extracted from healthy young leaflets of slightly opened leaf frond. One (1) gram of fresh leaf samples was homogenized using Liquid Nitrogen and the genomic DNA was isolated following a CTAB method (Doyle and Doyle 1987), modified with the addition of 2% polyvinylpyrilidone (PVP) in the extraction buffer. The palm is a selection in the ‘CATD’ plantation block maintained at the PCA Genebank in San Ramon, Zamboanga, Philippines. This specific variety of coconut was identified for genome sequencing due to its simple genome and low heterozygosity as ‘Dwarf’ coconut ecotype, and as a core parental genotype of PCA’s recommended hybrids. Selection was made in consultation with the PCA coconut breeders (Galvez *et al.* 2018).

Total gDNA samples were processed and assessed for the required quality and quantity for next-generation sequencing (NGS) analysis. The samples had an Invitrogen Qubit fluorometer (Thermo Fisher Scientific, Massachusetts) reading of 189 ng/uL and Nanodrop (Thermo Fisher Scientific, Massachusetts) OD 260/280 reading of 1.863. Processed gDNA samples of ‘CATD’ were shipped for outsourced Illumina Miseq sequencing to the Cornell sequencing facility, while the PacBio SMRT sequencing was serviced by the Cold Spring Harbor Laboratory. For Chicago sequencing, processed leaf tissue of the same coconut ‘CATD’ palm was shipped to the service provider (Dovetail Genomics LLC, CA).

### Illumina Miseq sequencing, PacBio SMRT sequencing and hybrid assembly

Using Illumina Miseq paired-end sequencing, a total of 109 Gb of paired-end fastq files were produced. With these data, the genome size of coconut was estimated by k-mer distribution through the KmerGenie program (Chikhi and Medvedev 2014). Using the String Graph Assembler (SGA; Simpson and Durbin 2012) sub-program ‘sga preprocess’, low quality bases below 30 were trimmed. After evaluation, the reads were indexed and the sequencing errors were corrected using ‘sga index’ and ‘sga correct’ sub-programs, respectively. The corrected read output of ‘sga correct’ was re-indexed and used as an input file for ‘sga filter’. The short-read data set was assembled using SPARSE assembler (Ye *et al.* 2012) with a k-mer length of 101 and filtered with length at least 1000 bp.

Pre-assembled short read NGS contigs were then used to correct and derive the compact representation of the 15x Pacific Bioscience Single-Molecule Real-Time (SMRT) long reads using the DBG2OLC hybrid assembler (Ye *et al.* 2016). The DBG2OLC assembler was used to assemble the raw PacBio SMRT sequence data, with the Illumina Miseq contig sequence assembly utilized as anchor for error correction. The overlap and consensus step were executed with the following parameters: k-mer value: 19; adaptive kmer matching threshold: 0.0001; fixed k-mer matching threshold: 2; minimum overlap score between a pair of long reads: 8; removal of chimeric reads: allow. The quality of the resulting assembly was assessed by means of a local Perl script (https://github.com/aubombarely/GenoToolBox/blob/master/SeqTools/FastaSeqStats) and the Benchmarking Universal Single Copy Ortholog (BUSCO) program using the plant-specific database of 1440 genes (Simão *et al.* 2015).

### Cell-free Hi-C for assembly and Genome Organization (Chicago) library preparation and sequencing

Three Chicago libraries were prepared as described previously (Putnam *et al.* 2016). Briefly, for each library, ∼500ng of high molecular weight gDNA (mean fragment length = 75 kb) was reconstituted into chromatin *in vitro* and fixed with formaldehyde. Fixed chromatin was digested with DpnII, the 5′ overhangs filled in with biotinylated nucleotides, and then free blunt ends were ligated. After ligation, crosslinks were reversed and the DNA purified from protein. Purified DNA was treated to remove biotin that was not internal to ligated fragments. The DNA was then sheared to ∼350 bp mean fragment size and sequencing libraries were generated using NEBNext Ultra enzymes and Illumina-compatible adapters. Biotin-containing fragments were isolated using streptavidin beads before PCR enrichment of each library. The libraries were sequenced on an Illumina HiSeq Platform. The number and length of read pairs produced for each library was: 128 million, 2x151 bp for library 1; 182 million, 2x151 bp for library 2; 140 million, 2x151 bp for library 3. Together, these Chicago library reads provided 175.91 × physical coverage of the genome (1-50kb pairs).

### Scaffolding the assembly with HiRise

The input *de novo* hybrid assembly, 50X Illumina Miseq PE reads, and Chicago library reads were used as input data for HiRise, a software pipeline designed specifically for using proximity ligation data to scaffold genome assemblies (Putnam *et al.* 2016). Miseq reads and Chicago library sequences were aligned to the draft input assembly using a modified SNAP read mapper (http://snap.cs.berkeley.edu). The separations of Chicago read pairs mapped within draft scaffolds were analyzed by HiRise to produce a likelihood model for genomic distance between read pairs, and the model was used to identify and break putative misjoins, to score prospective joins, and make joins above a threshold. After scaffolding, shotgun sequences were used to close gaps between contigs.

### Gap-filling, assembly correction and quantitative assessment of genome scaffold assembly

Post-processing of the resulting scaffolds was done using the gap-filling function of the PBJelly software package (English *et al.* 2012). The PacBio SMRT sequence data were used to anchor and further improve the contiguity of the scaffolds and reduce the number of ambiguous base ‘N’. Further correction was achieved using PILON automated genome assembly improvement tool (Walker *et al.* 2014). The binary alignment map (BAM) file was generated using Bowtie2 (Langmead and Salzberg 2012) by mapping the pre-processed Illumina Miseq PE reads to the scaffold assembly; output of which was input data for PILON assembly correction. The quality of the resulting assembly was assessed through a local Perl script as previously described, as well as TopHat2 (Kim *et al.* 2013) alignment of quality trimmed (Trimmomatic v0.36; SLIDINGWINDOW: 5:30; LEADING:5; TRAILING:5; MINLEN:100; Bolger *et al.* 2014) RNA-seq reads (SRR1173229). The quality is further evaluated with the Benchmarking Universal Single Copy Ortholog (BUSCO) program using the plant-specific database OrthoDB consisting of 1440 genes (Simão *et al.* 2015).

### Structural and functional annotation

The complete set of contigs was submitted to RepeatMasker version 3.3.0 (Chen 2004; www.repeatmasker.org) to identify significant similarity to the repeats available at RepBase (Jurka *et al.* 2005) and repeat models that were constructed *de novo* by RepeatModeler which combines RECON (Levitsky 2004) and RepeatScout (Price *et al.* 2005) programs. Using the repeat-masked genome, protein-coding sequences were predicted via ab initio, cDNA/EST, and homology-based approaches and integrated into the MAKER annotation pipeline (Cantarel *et al.* 2008). For the ab initio approach, AUGUSTUS (Stanke *et al.* 2006) was trained using BRAKER1 (Hoff *et al.* 2016) and the RNA-Seq raw reads (SRR1173229) were mapped into the assembled genome using TopHat2 (Kim *et al.* 2013). SNAP (Korf 2004), on the other hand, was self-trained using publicly available coconut cDNA/EST. These sets of predictions were merged with cDNA and protein sequences stored in the NCBI GenBank for coconut, *Elaeis guineensis* and six (6) other model plant genome through BLASTx, BLASTn and exonerate alignments. The other plant genomes used as reference were *Musa acuminata*, *Oryza sativa*, *Elaeis guineensis*, *Ananas comosus*, *Zingiber officinale* and *Zea mays*. Gene models generated from MAKER annotation were functionally annotated using Interproscan 5 (Jones *et al.* 2014) and BLAST2GO (Conesa *et al.* 2005).

### Repeat analysis

LTRharvest (Ellinghaus *et al.* 2008) and LTR_FINDER (Xu and Wang 2007) were both used to identify the presence of the full-length long terminal repeat retrotransposons (LTR-RTs) in the assembled coconut genome. All identified LTR-RTs from each of the LTR-RT identification programs were used as an input to LTR_retriever (Ou and Jiang 2017) to generate a high-quality non-redundant LTR libraries, and to categorize them into two families (*i.e.*, gypsy and copia) in order to estimate the predicted insertion dates of various types of repeat sequences. The insertion dates for each specific LTR-RTs were estimated using the Jukes-Cantor model (Jukes and Cantor 1969) for noncoding sequences, and mutation rate of 1.3 × 10^−8^ mutations per site per year as previously proposed (Ma and Bennetzen 2004). The evolutionary history of non-redundant set of LTRs was investigated using the multiple sequence alignment of the individual LTR sequences by CLUSTALW (Thompson *et al.* 1994) followed by Maximum Likelihood method based on the Tamura-Nei model (Tamura and Nei 1993), with 1000 bootstrap replications and using the MEGA7 program (Kumar *et al.* 2016).

### Comparative genomics

Structural and temporal syntenic relationship was analyzed between the assembled dwarf coconut genome and published genomes of tall coconut (Xiao *et al.* 2017), date palm (Al-Mssallem *et al.* 2013) and oil palm (Singh *et al.* 2013) using the CoGe (Lyons and Freeling 2008) SynMap (Lyons *et al.* 2008) tool. The assembled genome and corresponding gene annotation of the dwarf coconut were privately uploaded to CoGe. To detect structural variation between these palm genomes, >1 Mbp genomic scaffolds each of the current dwarf coconut assembly, published tall coconut and date palm, and 16 pseudomolecules of oil palm were analyzed using the Syntenic Path Assembly in SynMap (BLAST algorithm - LAST (Kiełbasa *et al.* 2011), DAGChainer (Haas *et al.* 2004) – Relative Gene order, −D = 20, −A = 5, skip random/unknown chromosomes). The rate of synonymous substitution (Ks) values was also calculated (temporal calculation of synteny) for the detected syntelogous gene pairs using the Needleman-Wunch algorithm in the nwalign software (Needleman and Wunsch 1970) and CodeML of the PAML package (Yang 1997) both integrated in SynMap.

### Annotation for disease and insect resistance genes

Genome-wide resistance gene analogs (RGA) were identified in the generated gene models from genome annotation using RGAugury (Li *et al.* 2016) The RGAugury is an automated RGA prediction pipeline and target RGA candidates included Nucleotide Binding Site (NBS) and transmembrane-coiled-coil (TM-CC) containing proteins and membrane associated receptor-like kinase (RLK) and receptor-like proteins (RLP) families. The input protein sequences were initially filtered by BLASTp search against RGAdb database at E-value cut-off of 1e-5. The domain and motif of the initial set of candidate RGAs were characterized using the nCoils, phobius, pfam_scan and InterProScan third-party programs.

The FASTA amino acid sequences of all the identified candidate RGAs were used to construct a phylogenetic tree. Multiple Sequence Alignment (MSA) of the RGA sequences was performed using the CLUSTALW program (Thompson *et al.* 1994) with the following parameters: Gap Opening Penalty: 10; Gap Extension Penalty: 0.2. The phylogeny of these aligned sequences was reconstructed using Maximum Likelihood statistical method using IQ-TREE (Nguyen *et al.* 2015) with best-fit substitution model selected through ModelFinder (Kalyaanamoorthy *et al.* 2017). Based on the Bayesian Information Criterion (BIC) of the models, JTT amino acid substitution model (Jones *et al.* 1992) with empirical codon frequencies (+F) and FreeRate (+R9) rate heterogeneity across sites (Yang 1995; Soubrier *et al.* 2012) was used to generate the tree. The resulting phylogenetic tree was validated with 1000 replicates of ultrafast bootstrapping (Hoang *et al.* 2017) and SH-aLRT (Guindon *et al.* 2010) tests.

### Annotation for oil biosynthesis and drought-response genes

The cDNA sequences in FASTA format of oil biosynthesis genes such as 1-acyl-sn- glycerol-3-phosphate acyltransferase (LPAAT) and phospholipase D from coconut, and 3-hydroxyisobutyryl-CoA hydrolase-like and pyruvate dehydrogenase E1 β subunit from *Elaeis guineensis*, were downloaded from NCBI (https://www.ncbi.nlm.nih.gov/). These sequences were used as the query in BLASTn alignments to the coconut ‘CATD’ genome. All the drought-related proteins available at DroughtDB (Alter *et al.* 2015; http://pgsb.helmholtz-muenchen.de/droughtdb/) were downloaded. BLASTp analysis on the GO-assigned gene families was performed and subsequently filtered with an e-value of 0.0, minimum percent (%) identity of 50 and minimum alignment length of 100. The drought-related gene homologs in coconut were functionally annotated using BLAST2GO (Conesa *et al.* 2005) with default settings for the mapping and annotation step to reveal gene ontology terms.

### DNA marker design for coconut breeding applications

Genome-wide SSR mining, statistical classification and marker generation were done using the GMATA package (Wang and Wang 2016). The SSR loci in the assembled genome were identified using the following parameters in the package: minimum repeat motif length: 2 nucleotides; max repeat motif length: 10 nucleotides; minimum repeat times: 5. The assembled genome and identified SSR loci used as input data to design SSR markers using Primer3 software integrated in the GMATA package. The following settings were used: minimum amplicon size: 120 bp; maximum amplicon size: 400 bp; optimal annealing temperature: 60°; flanking sequence length: 400; maximum template length: 2000.

Variations (SNPs and InDels) between the assembled reference ‘Dwarf’ coconut genome and the reference tall coconut genome were detected following the GATK Best Practices workflow (Van Der Auwera *et al.* 2013). The Hainan Tall WGS sequence read archive (SRA) files were downloaded from NCBI (SRR5273820, SRR5273822). The downloaded paired-end (PE) reads were pre-processed using Trimmomatic v0.36 (Bolger *et al.* 2014) using the following parameters: SLIDINGWINDOW: 5:30; LEADING:5; TRAILING:5; MINLEN:50. Hainan Tall pre-processed PE reads were mapped on the repeat-masked ‘Catigan Green Dwarf’ genome using Burrows-Wheeler Aligner tool (bwa; Li 2013) with default settings. Picard toolkit (http://broadinstitute.github.io/picard/) was used to generate a sorted de-duplicated binary alignment map (BAM) file from the sequence alignment map (SAM) file from the read mapping step. The read mapping artifacts were minimized by local realignment around indels using GATK v3.8 RealignnerTargetCreator and IndelRealigner commands. To correct the bias of the per-base estimate of error generated by the sequencing platform, base quality score recalibration (BQSR) was performed using GATK BaseRecalibrator and PrintReads commands. Variants were called by the GATK HaplotypeCaller through setting the output mode to EMIT_VARIANTS_ONLY, and calling confidence threshold (stand_call_conf) to 30. VCFTools (Danecek *et al.* 2011) was used to filter the high-quality SNP calls and to compute basic transversion/transition statistics.

### Data availability

The assembled sequence data of the ‘Catigan Green Dwarf’ coconut variety has been submitted to the DDBJ/ENA/GenBank database under accession number QRFJ00000000. The version described in this paper is version QRFJ01000000. Figure S1 shows the basic statistics and per base sequence analysis result of the pre-processed Illumina Miseq short read sequences. Figure S2 depicts the genome size estimation of the CATD coconut variety based on the generated homozygous k-mer peak. Figure S3 shows the BUSCO analysis of the constructed genome assembly based on 1440 plant-specific genes in the OrthoDB database. Figure S4 shows the distribution and characterization of the repeat elements characterized in the coconut ‘CATD’ genome draft assembly. Figure S5 presents the molecular phylogenetic relationships of the core LTR-RT in the coconut ‘Catigan Green Dwarf’ (CATD). Figure S6 shows the predicted gene ontology (GO) distribution of the genes in coconut genome. Figure S7 predicts the genome-wide identification and characterization of resistance gene analogs (RGA) in the coconut genome. Figure S8 shows the distribution of the drought-response gene homologs classified based on characterized biological function in coconut. Figure S9 presents the proportion of SSR motif found in the current assembly of the ‘CATD’ genome. Figure S10 depicts the genome-wide occurrence of top paired-motif in coconut (‘CATD’) SSRs. Table S1 compares the assembly and quality statistics of ‘CATD’ coconut genome *vs.* HAT coconut genome, and other closely related sequenced genomes. Table S2 compares the quality of genome annotation of the assembled ‘CATD’ coconut genome with the annotated HAT genome, and other closely related sequenced genome. Table S3 list of genome-wide transcription factors and other transcriptional regulators identified in the predicted genome models of ‘CATD’ genome. Table S4 presents the BLASTn output of the alignment of oil biosynthesis cDNA sequences in the coconut ‘CATD’ genome. Table S5 lists the developed SSR markers physically linked to economically important traits in coconut. Data S1 lists the LTR-RT found in regions of the dwarf coconut genome with estimated insertion dates. Data S2 shows the dag-chainer output file of ‘CATD’-date palm alignment. Data S3 contains the FASTA sequences of the core LRT-RT in coconut genome. Data S4 contains the BLASTp alignment result of the coconut predicted gene models to DroughtDB proteins. Data S5 lists the genome-wide SSR markers designed in coconut. Supplemental material available at FigShare: https://doi.org/10.25387/g3.8209889.

## Results and Discussion

### Coconut ‘CATD’ de novo genome sequence assembly

The whole genome sequence (WGS) of ‘Catigan Green Dwarf’ (CATD) coconut variety was generated using Illumina Miseq, PacBio SMRT and Dovetail Chicago sequencing technologies. The ‘CATD’ variety of coconut was chosen being a Dwarf ecotype, hence highly homozygous and simpler genome architecture, also believed to have a smaller genome size and is the best parent Dwarf variety of Philippine Coconut Authority’s recommended hybrids for coconut oil. The ‘CATD’ genome has an estimated size of 2.15 Gbp based on the generated homozygous k-mer peak using the preprocessed Illumina Miseq short reads (Figure S1; Figure S2). The estimated genome size of coconut is higher than what has been reported in date palm (660 Mbp; Al-Mssallem *et al.* 2013), African oil palm (1.8 Gbp; Singh *et al.* 2013), and American oil palm (1.8 Gbp; Singh *et al.* 2013). The calculated ‘CATD’ genome size, however, is lower than the estimated genome size (2.42 Gbp) of the recently sequenced ‘Hainan Tall’ (HAT) coconut variety (Xiao *et al.* 2017) by 280 Mbp. Freitas Neto *et al.* (2016) reported that the genome size variation using flow cytometry method among 14 coconut ‘Tall’ and ‘Dwarf’ varieties is statistically small, and concluded that ‘Dwarf’ types did not really evolve from ‘Tall’ types as previously hypothesized (Perera *et al.* 2016). We suggest otherwise, since there is a large difference between the genome sizes based on k-mer peak analysis between ‘Hainan Tall’ (Xiao *et al.* 2017) and ‘Catigan Green Dwarf’ (this paper) whole genome sequence data. Nevertheless, we concur that this inference has to be validated with more sequence data of several genome samples of other ‘Dwarf’ and ‘Tall’ coconut varieties to determine the statistical significance of this finding.

The pre-processed reads from Illumina Miseq sequencing were assembled as described (Materials and Methods). The analysis returned an assembled genome of a total length of 1.59 Gbp covering 73.9% of the estimated genome and N50 value of 5,247 bp (Table 1). The large size and complexity of the coconut genome due to repeat elements explain the observed low N50 and genome coverage of the assembled Illumina Miseq short reads.

**Table 1:** Statistical summary of the ‘Catigan Green Dwarf’ (CATD) coconut assembly using various sequencing technologies and corresponding bioinformatics pipelines

PARAMETERS	SPARSE (ILLUMINA MISEQ)	SPARSE + DBG2OLC (ILLUMINA MISEQ + PACBIO SMRT)	HIRISE PIPELINE + PBJELLY (DRAFT ASSEMBLY + DOVETAIL CHICAGO)
**Assembly Summary**			
Genome Coverage	73.9%	88.3%	97.6%
Sequence Count	482,724	25,020	7,998
Total Length	1.59 Gbp	1.9 Gbp	2.102 Gbp
N50	5,247 bp	119 kbp	570,487 bp
Longest Sequence	57,454 bp	1,725,761 bp	8,779,653 bp
Shortest Sequence	801 bp	906 bp	1,912 bp
Average Length	3,295.14 bp	76,510 bp	570,487 bp
GC Level	—	—	37.64%
N Content	—	—	0.285%
Number of Gaps	—	—	12,106
Complete BUSCOs	—	—	1322 (91.8%)
Alignment Rate (‘CATD’ Illumina Miseq WGS)	—	—	96.96%
Alignment Rate (Quality-trimmed RNAseq reads - SRR1173229)	—	—	95.7%
**Annotation Summary**			
Number of gene models	—	—	34,958
Average gene length			7724.72 bp
Average exon length	—	—	267.36 bp
Average intron length			1448.73 bp
Average number of exons per gene	—	—	5.34
Average number of introns per gene	—	—	4.34
Average protein length	—	—	373.18
Complete BUSCOs	—	—	85.3

To improve the assembly associated with large genome size and high complexity, the same palm sample of coconut ‘CATD’ was further sequenced using the long read NGS technology of PacBio SMRT. The hybrid assembly consisting of assembled long reads corrected with processed short reads, generated a total of 25,020 contigs with N50 value of 119 kbp and representing a total length of 1.9 Gbp of the coconut genome. As reported in this paper, the final genome scaffold assembly of coconut ‘CATD’ has a total length of 2.1 Gbp consisting of 7,998 scaffolds and N50 of 570,487 bp, achieved using Dovetail Chicago sequencing, gap-filling and error correction (Table 1).

### General characteristic and quality of the final genome scaffold assembly

More than 96.96% of the short reads were mapped to the scaffold to estimate the coverage of the current genome assembly for coconut ‘CATD’. The quality-trimmed RNA-seq reads (SRR1173229; Rajesh *et al.* 2016) were likewise aligned on the assembly to achieve an overall mapping rate of 95.7%. This suggests the degree of capture of expressed transcripts in the assembled genome (Table 1). Based on Benchmarking Universal Single Copy Ortholog (BUSCO) analysis, the genome contains 91.8% complete and 3.1% fragmented single copy orthologs (SCOs) in reference with 1440 plant-specific genes in the OrthoDB database (Simão *et al.* 2015). This further supports the high quality and completeness of the assembled genome (Figure S3).

The quality of the current ‘CATD’ scaffold assembly is compared with the genome assembly of coconut ‘Hainan Tall’ (HAT; Xiao *et al.* 2017) and other close relative palms. In terms of assembly statistics and quality evaluation results (Table S1), the ‘CATD’ genome has higher contig N50 and scaffold N50 in comparison with ‘HAT’ by 46.36 kbp and 151.93 kbp, respectively. The scaffold N50 of ‘CATD’, however, is lower than that of the E5-build of African oil palm (Singh *et al.* 2013). In terms of number of SCOs included in the genome assembly, the ‘CATD’ assembly has the highest number of complete orthologs based on BUSCO analysis data output. This demonstrates that the current scaffold assembly of ‘CATD’ has covered most of the ‘Dwarf’ *Cocos nucifera* L. genome and gene units. The ‘CATD’ genome assembly can therefore provide a good reference for various applications in coconut such as re-sequencing projects, functional genomics studies, and gene mining/DNA marker development.

### ‘CATD’ genome features and annotated gene models

The coconut ‘CATD’ genome, as currently assembled, is composed of 37.74% GC. Repeats remain to be the technical challenge in the assembly as with other complex genomes. As the major genomic elements, detailed characterization of the repeats can provide invaluable information to refine the process of assembly and annotation. A total of approximately 1.648 Gbp of sequence was identified as unclassified and interspersed repeats representing about 78.33% of the total assembled genome (Figure S4). This observed composition of repeat elements is comparable to the repeat content reported in the maize genome (Schnable *et al.* 2009). Furthermore, as expected in plants, retroelements were the most common mobile elements found in the assembly and highly represented by LTR elements (60.26%). Other repeats identified are satellites (1.4 Mbp; 0.07%), simple repeats (19.5 Mbp; 0.93%) and low complexity repeats (2.5 Mbp; 0.12%) (Figure S4).

Being the most abundant type of interspersed repeat sequence in the ‘CATD’ genome, the full-length long terminal repeat (LTR) retrotransposons were analyzed and characterized. A total of 3,670 LTR elements were determined, and classified as Copia (922), Gypsy (742) and unknown elements (2066) (Data S1). Although the function of the LTR-RT in the host genome is still not well understood, their existence is highly recognized to play an important role in the maintenance of the structure of the chromatin as well as to regulate the host’s machinery for gene expression (Zhao and Ma 2013). Furthermore, investigations on the recent insertion of transposable elements can provide insight on the evolutionary events during genome speciation (Liu and Yang 2014). In the current assembly, the age and molecular diversity of the identified repeat elements were investigated to infer the dynamics of these LTR-RTs during the evolution of the coconut genome.

**Figure 1:**
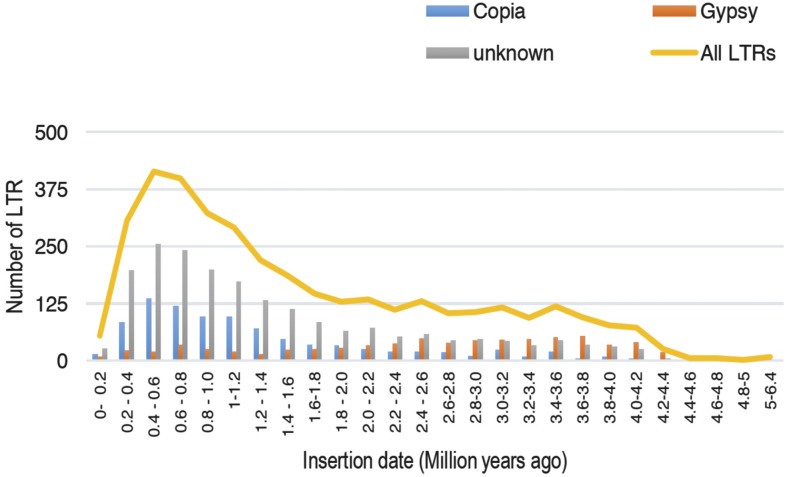
Insertion time distributions of intact LTR in the ‘CATD’ coconut genome estimated using the Jukes-Cantor model (Jukes and Cantor 1969) for noncoding sequences, and mutation rate of 1.3 × 10^−8^ mutations per site per year (Ma and Bennetzen 2004).

The approximate insertion dates of each Copia, Gypsy and Unknown LTR elements in the ‘CATD’ coconut genome were determined (Figure 1). This was estimated by comparing the 5′ LTR and 3′ LTR of each LTR-RT using the Jukes-Cantor model (Jukes and Cantor 1969) for non-coding sequences, and based on a proposed mutation rate of 1.3 × 10^−8^ mutations per site per year (Ma and Bennetzen 2004). Insertion data shows that 3,552 (96.79%) elements proliferated in the last 4 million years, and only 774 (21%) elements were inserted in the last 600,000 years. A total of 54 (1.5%) LTR elements could be aged between 0-200,000 years ago indicating that retrotransposon activity in coconut is already declining. Ancient insertions of LTR retrotransposons (2 to 6.4 million years ago) were characterized to be highly predominated by a high activity of Gypsy elements, while a high rate of insertion of Copia superfamilies could be deduced to have occurred during the last 2 million years. Furthermore, results provided evidence that a rapid burst of total LTR elements happened in coconut around 400,000 years ago, which coincides with the recorded era of earth’s transition from glacial (ice age) to interglacial period (Termination V – 430,000 years ago) characterized by changes in temperature and greenhouse gasses (Augustin *et al.* 2004). The selection pressure imposed by harsh changes in the environment would have shaped coconut gene diversity at the genome-sequence level including the composition of repeat elements.

A non-redundant database consisting of 112 core LTR-RT sequences is created for the ‘CATD’ genome and classified into 47 Gypsy, 26 Copia and 39 Unknown LTR-RT elements (Data S3). Based on the sequence homology analysis of these coconut repeat elements against characterized plant genomic elements, ‘CATD’ Gypsy was found to be more diverse than its Copia elements. The molecular phylogenetic tree of the core LTR-RT elements of ‘CATD’ was also built using the maximum likelihood clustering method (Tamura and Nei 1993) with 1,000 bootstrapping, wherein four (4) major homology clusters are apparent (Figure S5). The impact of the evolutionary pattern of individual LTR-RT element on the extent of biological diversity of ‘CATD’ could not be inferred yet with the current data. Nonetheless, the identification and characterization of these LTR-RT elements in the host ‘CATD’ genome is the first step toward the elucidation of the possible mechanism of interaction between repeat elements and functional genes in coconut.

### ‘CATD’ protein-coding genes

A total of 34,958 protein-coding gene models were predicted in the ‘CATD’ genome assembly on the basis of ab initio and evidence-based methods (Table 1). Structural annotation returned an average number of 5.34 exons per gene based on the gene structure of all identified protein-coding sequences. The quality of genome annotation was assessed using 1440 plant BUSCOs by checking the proportion of single copy orthologs (SCO) that could be predicted and annotated structurally in the assembly (Table S2). Results showed that the ‘CATD’ genome has a more complete set of annotated genes (85.3%) as compared to that of ‘Hainan Tall’ annotated genes (81.2%), date palm PDK30 build (57.5%) and African oil palm E5 build (42.4%). However, the complete BUSCO in date palm DPV01 build (94%) is higher than that of the ‘CATD’ coconut annotated genes.

More than 74% of the gene models contain homologs in the SwissProt/UniProt non-redundant database (E-value = 1e−6). About 54.2% (18,950 annotated genes) of the annotations could also be assigned to a gene ontology (GO) catalog and 92.2% (32,261) with characterized InterProScan functional domains. Based on the complete scan or genome-wide functional annotation of the predicted genes in the ‘CATD’ genome, the most InterProScan sites in coconut could be characterized as serine/threonine protein kinase active site, protein kinase ATP binding site, and IQ motif EF-hand binding site (Figure S6). Direct counts of the identified GOs also revealed that majority of the biological processes (BP) are involved in metabolic processes such as protein phosphorylation, oxidation-reduction and regulation of transcription. The molecular functions (MF) of the genes in ‘Dwarf’ coconut, as in the current ‘CATD’ genome annotation, could be mainly associated to binding (protein, ATP, DNA) followed by catalytic and transporter activities. Furthermore, direct counts of GOs for the cellular component (CC) showed that most coconut genes are integral components of the cell membrane.

### Comparative genomics of the dwarf coconut genome

**Figure 2:**
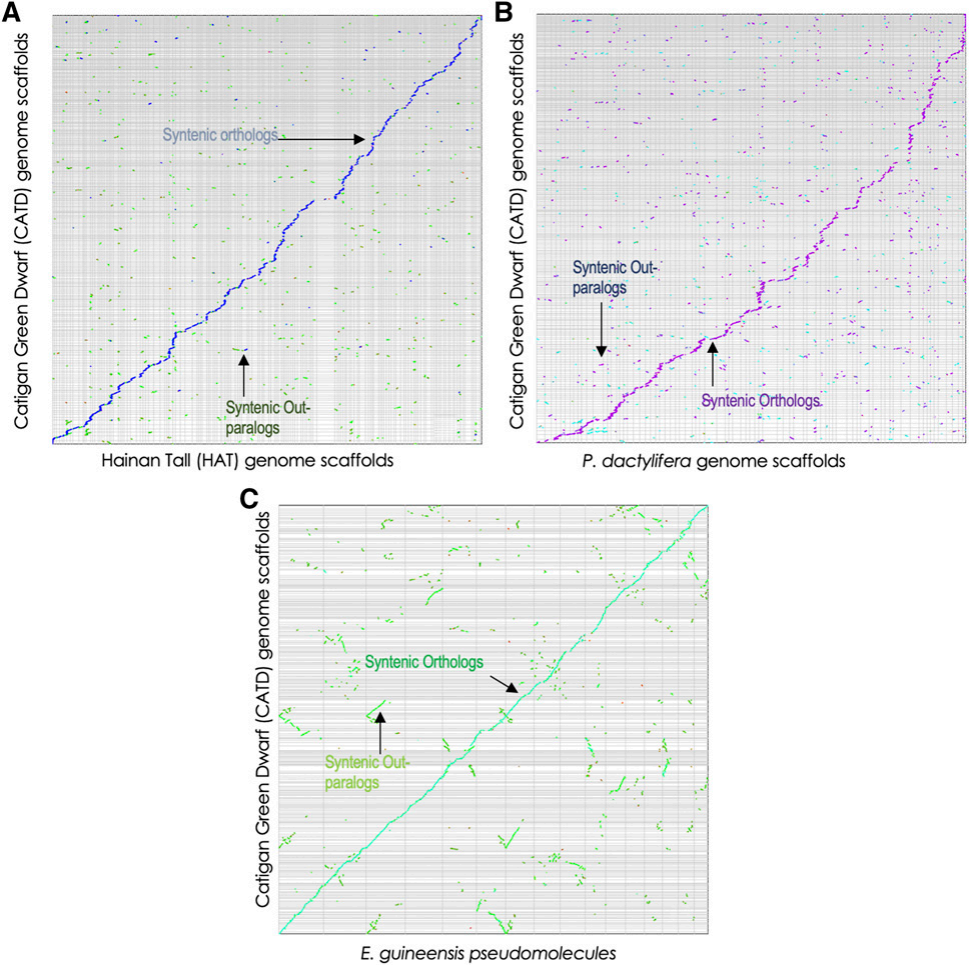
Syntenic dotplot between dwarf coconut var. Catigan Green dwarf (CATD) and tall coconut var. Hainan Tall (2a), CATD and date palm (*P. dactylifera*) (2b), and CATD and oil palm (*E. guineensis*; 2c). The dotplot axis matrix is in nucleotides with square dotplot axes relationship. The scaffolds in the y-axis of both (a) and (b) are arranged in the same manner by order of scaffold number. Scaffolds in the y-axis of (c) are sorted based on the Syntenic Path Assembly (SPA) using oil palm pseudomolecules as reference. The figures are generated using the Legacy Version of CoGe SynMap tool (Lyons *et al.* 2008).

As the major members of the family *Arecaceae*, characterization of the genomic variations/synteny among coconut, oil palm and date palm will provide insights on the evolutionary pattern of divergence within the palm family; at least structurally at the genome sequence level. Genome sizes among these palms vary significantly (Singh *et al.* 2013; Al-Mssallem *et al.* 2013; Xiao *et al.* 2017). The basic chromosome number (n) between the coconut (n = 16; Batugal *et al.* 2009) and oil palm (n = 16; Madon *et al.* 2005; Maria *et al.* 1995) is conserved but not against date palm (n = 18; Al-Salih and Al-Rawi 1987; Mathew *et al.* 2014). The whole genome sequence assembly of oil palm (Singh *et al.* 2013) and date palm (Al-Mssallem *et al.* 2013) enabled the identification of oil palm’s duplicated genomic regions that are in synteny with unique scaffolds of date palm. This provides structural evidence that the progenitor of both oil and date palms is a polyploid species (Singh *et al.* 2013). Likewise, we used a syntenic path assembly comparison to analyze synteny between the assembled ‘CATD’ genome to previously published genomes of the other coconut type (Xiao *et al.* 2017) and closely related species (Singh *et al.* 2013; Al-Mssallem *et al.* 2013; Figure 2). A total of 543 genome scaffolds of the dwarf coconut are found to be of high level of synteny to 974 scaffolds of the earlier published tall-type coconut (Xiao *et al.* 2017) with pairwise syntelogs of 23,207. Meanwhile, the whole genome alignment of the dwarf coconut genome to the genomes of date palm (Al-Mssallem *et al.* 2013) and oil palm (Singh *et al.* 2013) revealed 27,549 and 29,587 syntelogs, respectively. This further validates the quality/high-degree of gene completeness of the assembled dwarf coconut genome, and its close evolutionary relationship with the other palm species.

The whole genome alignment of the assembled dwarf coconut against the published tall coconut revealed a high degree of synteny, which is expected since both are genomes of the same crop species (Figure 2a). However, syntenic orthologs and out-paralogs are found to be duplicated and widely distributed among their genomic scaffolds. This finding is suggestive of possible chromosome segment duplications, transposition events, and other evolutionary genomic changes between the two genomes of different coconut types. Similar results are also obtained between the dwarf coconut and date palm genomic alignment. Segmental duplications of 602 scaffolds of date palm are found in synteny across 561 genomic scaffolds of coconut (Data S2). The synteny analysis provides an overview of possible mechanism of genomic changes during the evolution of the two coconut types (Dwarf coconut and Tall coconut), and divergence between coconut and date palm. However, this evolutionary genome analysis is still inconclusive since the current builds of the three (3) genome assemblies - dwarf coconut, tall coconut, and date palm - are yet at the scaffold level.

Interestingly, the whole genome alignment between the dwarf coconut and oil palm provided clearer insights to their dynamic chromosomal changes during the course of evolution (Figure 2c). Large portions of the oil palm genome are in synteny with several other genomic scaffolds of the dwarf coconut demonstrating possible chromosomal duplications and fractionation followed by series of rearrangements and inversions. This result suggests that the coconut palm arose from the re-diploidization of a common polyploid ancestor, which also supports previous report and hypothesis between oil palm and date palm (Singh *et al.* 2013). This evolutionary event might have triggered the varying degree of genome expansions among the members of the family *Arecaceae*.

The coding sequence divergence of the dwarf coconut (CATD) between the genomes of tall coconut (HAT), oil palm and date palm was measured by synonymous changes (Ks). Overall, the mean Ks between CATD and HAT genomes (Ks_CATDvsHAT_ = -1.0148) is less than that of CATD against oil palm (Ks_CATDvsOilPalm_= -0.2774) and CATD *vs.* the date palm (Ks_CATDvsDatePalm_= -0.3426). The coding gene sequences between the two coconut types are therefore very well-conserved. Moreover, the data (Ks_CATDvsHAT_ < Ks_CATDvsOilPalm_ < Ks_CATDvsDatePalm_) further supports previous evidences and reports that the coconut palm is more related to oil palm than date palm (Huang *et al.* 2013; Xiao *et al.* 2017).

**Figure 3:**
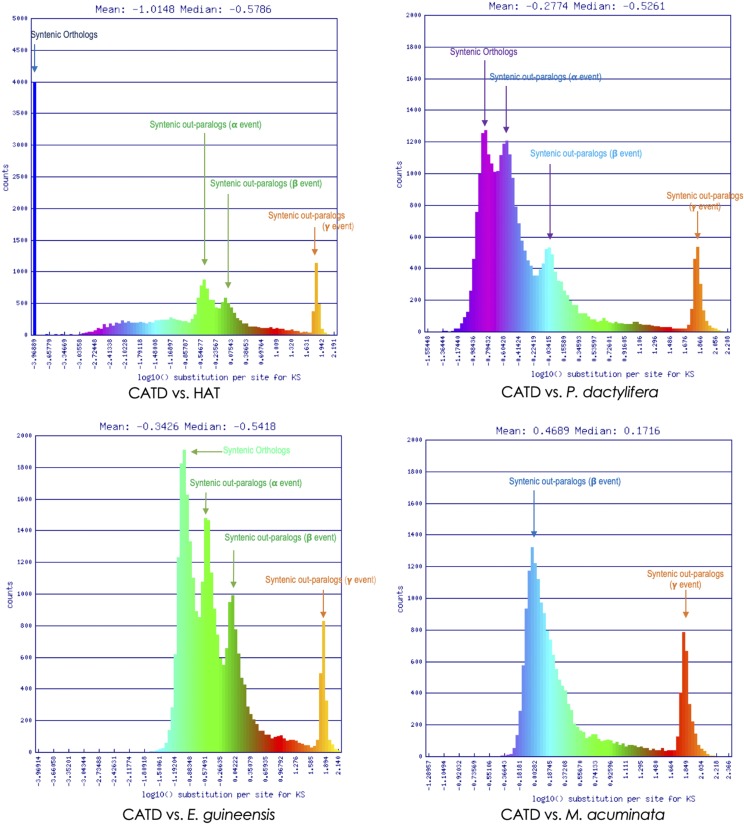
Histogram depicting the synonymous rate change of syntenic gene pairs between dwarf coconut and other closely related sequenced genomes. The syntenic gene pairs were identified by DAGChainer, and colored based on their synonymous substitution rate as calculated by CodeML of the CoGe SynMap tool (Lyons *et al.* 2008). Syntenic regions derived from speciation (orthologs) from shared whole genome duplication events (α, β and γ) are also labeled.

Four (4) distinct peaks are detected on the constructed histogram plot of Ks values, which represent three (3) rounds of whole genome duplication events (WGD; α, β and γ) shared by coconut, oil palm and date palm (Figure 3). This number of WGD events was also observed in banana (*Musa acuminata*), a close relative of the palm family (D’Hont *et al.* 2012). However, only two (2) WGD events (β and γ) were detected when the Ks values of the coding gene sequences of dwarf coconut and banana were calculated and plotted on a histogram. Thus, we propose that the *Arecaceae* shares a common WGD β and γ events with the *Musa* lineage but independent WGD α event, which probably happened after the divergence of *Arecaceae* and *Musa*.

### Transcription factors and other transcriptional regulators

A total of 4,124 (10% of the total predicted gene models) regulatory genes were identified in the ‘CATD’ dwarf coconut genome, which is higher than that in carrot (3,267), tomato (3,209) and rice (3,203) regulatory genes. There are 99 unique families of regulatory genes that are mostly classified as transcription factor (TF) family type. Other types of protein families involved in gene regulation in dwarf coconut, as characterized in the ‘CATD’ genome, are transcription factor interactor and regulator, chromatin regulator, chromatin remodeling and transcription regulator, and lipid-binding proteins (Table S3).

The Cys2His2 (C2H2) transcription factor family is the most over-represented TF family in the coconut ‘CATD’ genome with 565 genes or 13.7% of the total TF genes and other transcriptional regulators. The C2H2 TF is also higher in coconut compared to the identified C2H2 genes in *Arabidopsis* (176 genes; Englbrecht *et al.* 2004), rice (189 genes; Agarwal *et al.* 2007), foxtail millet (124 genes; Muthamilarasan *et al.* 2014), and poplar (109 genes; Liu *et al.* 2015). C2H2-type zinc fingers are widespread DNA binding motifs in eukaryotic transcription factors. The stability of the structure of the zinc finger is achieved by the interaction of two cysteine and two histidine residues located in certain positions of the zinc element. The number of zinc fingers in a single protein is highly diverse, while single-zinc finger containing transcription factors are only present in the plant kingdom. Aside from the ability of the zinc fingers to act as DNA binding proteins, they can also affect other gene networks by interacting with other proteins as well as RNA molecules therefore regulating RNA metabolism and other biological phenomena (Razin *et al.* 2012). Zinc finger has a broad-range of biological function in plants including plant growth and development (trichome development, seed germination, floral organogenesis, secondary metabolism and cell wall structure), and responses to biotic (pathogen defense) and abiotic stresses (cold, drought, salinity, mechanical) (Kim *et al.* 2006; Dinneny *et al.* 2006; Wu *et al.* 2008; Al-Ghazi *et al.* 2009; Huang *et al.* 2009; Sun *et al.* 2010; Feurtado *et al.* 2011; Gourcilleau *et al.* 2011; Fan *et al.* 2015; Liu *et al.* 2015).

### Oil biosynthesis genes

Medium chain fatty acids (MCFAs) and long-chain fatty acids comprise around 83.92% of the total composition of copra oil of which lauric acid (C12) is the most predominant (Padolina *et al.* 1987; Laureles *et al.* 2002). Liang *et al.* (2014) reported the roles of 1-acyl-sn-glycerol-3-phosphate acyltransferase (LPAAT), phospholipase D, acetyl-CoA carboxylase carboxyltransferase beta subunit, 3-hydroxyisobutyryl-CoA hydrolase-like and pyruvate dehydrogenase E1 β subunit in the fatty acid biosynthesis in coconut using KEGG analysis from suppression subtractive hybridization (SSH) experiment. The coconut cDNA sequences of LPAAT and phospholipase D are already available in the public repository but none has been reported for 3-hydroxyisobutyryl-CoA hydrolase-like and pyruvate dehydrogenase E1 β. Thus, for this later 2 genes, the cDNA sequences from *E. guineensis* were used to characterize the homologs in CATD coconut. Conversely, the gene sequence for acetyl-CoA carboxylase carboxyltransferase beta subunit has already been characterized; thus, not included in this study.

All cDNAs have a corresponding BLASTn hit in the ‘CATD’ genome except for the *Elaeis* cDNA sequence of 3-hydroxyisobutyryl-CoA hydrolase-like protein, which is probably due to high sequence divergence of this gene between the two palm genomes (Table S4). The coconut gene sequences for LPAAT and pyruvate dehydrogenase E1 β were found to be in one (1) copy. On the other hand, two (2) homologous genes of phospholipase D alpha 1-like protein are located in two separate genome scaffolds.

LPAAT is a major gene responsible for the high accumulation of lauric acid in coconut as previously demonstrated in the genetic transformation experiments of this gene in *Brassica napus* (Knutzon *et al.* 1999). The CATD coconut LPAAT gene is characterized to be 28,344 bp in length, shorter than the gene sequence of African oil palm by 10,487 bp (Singh *et al.* 2013). Analysis of its gene structure in coconut should provide an effective reference to potentially target gene editing and variation screening toward improved copra oil quality among others enhanced mutant phenotypes. Manohar *et al.* (2018) reported that EcoTILLING of this gene in 48 coconut varieties has revealed a natural SNP between West African Tall (WAT) and Aguinaldo Tall (AGDT) varieties. Opportunity in coconut breeding for improved coconut oil can be directed using these coconut varieties, and in reference with the whole genome sequence of coconut.

### Resistance gene analogs

Genome-wide resistance gene analogs (specifically NBS-LRR and TM-CC) were characterized from the collection of predicted gene models. The conserved domains and motifs as characterized in plant resistance gene analogs (RGA) were analyzed in the gene models of coconut ‘CATD’ genome. These were nucleotide binding sites (NBS-ARC), leucine rich repeats (LRR), transmembrane (TM), serine/threonine and tyrosine kinase (STTK), lysine motif (LysM), coiled-coil (CC) and Toll/Interleukin-1 receptor (TIR). Encompassing the whole genome, 340 RGA genes were identified and classified into six (6) major classes as follows: (a) 90 genes encoding for CC-NBS-LRR (CNL); (b)16 genes encoding for CC-NBS (CN); (c) 2 genes encoding for TIR-NBS (TN); (d) 34 genes encoding for NBS-LRR (NL); (e) 5 genes encoding for TIR-unknown domain (TX); and (e) 192 genes encoding for transmembrane-coiled coil (TM-CC). In the current genome annotation of ‘CATD’, none are detected for genes encoding TIR-NBS-LRR (TNL) and other NBS variants (Figure S7). The absence of TNL is expected since it was previously hypothesized that TNLs have never evolved in monocots (Tarr and Alexander 2009). The majority of the predicted RGAs belong to the TM-CC class, which is higher in number as reported in corn (161 TM-CC), sorghum (128 TM-CC), rice (158 TM-CC) and banana (138 TM-CC); but lower than the 218 TM-CC RGA genes predicted in *Panicum virgatum* (Li *et al.* 2016).

**Figure 4:**
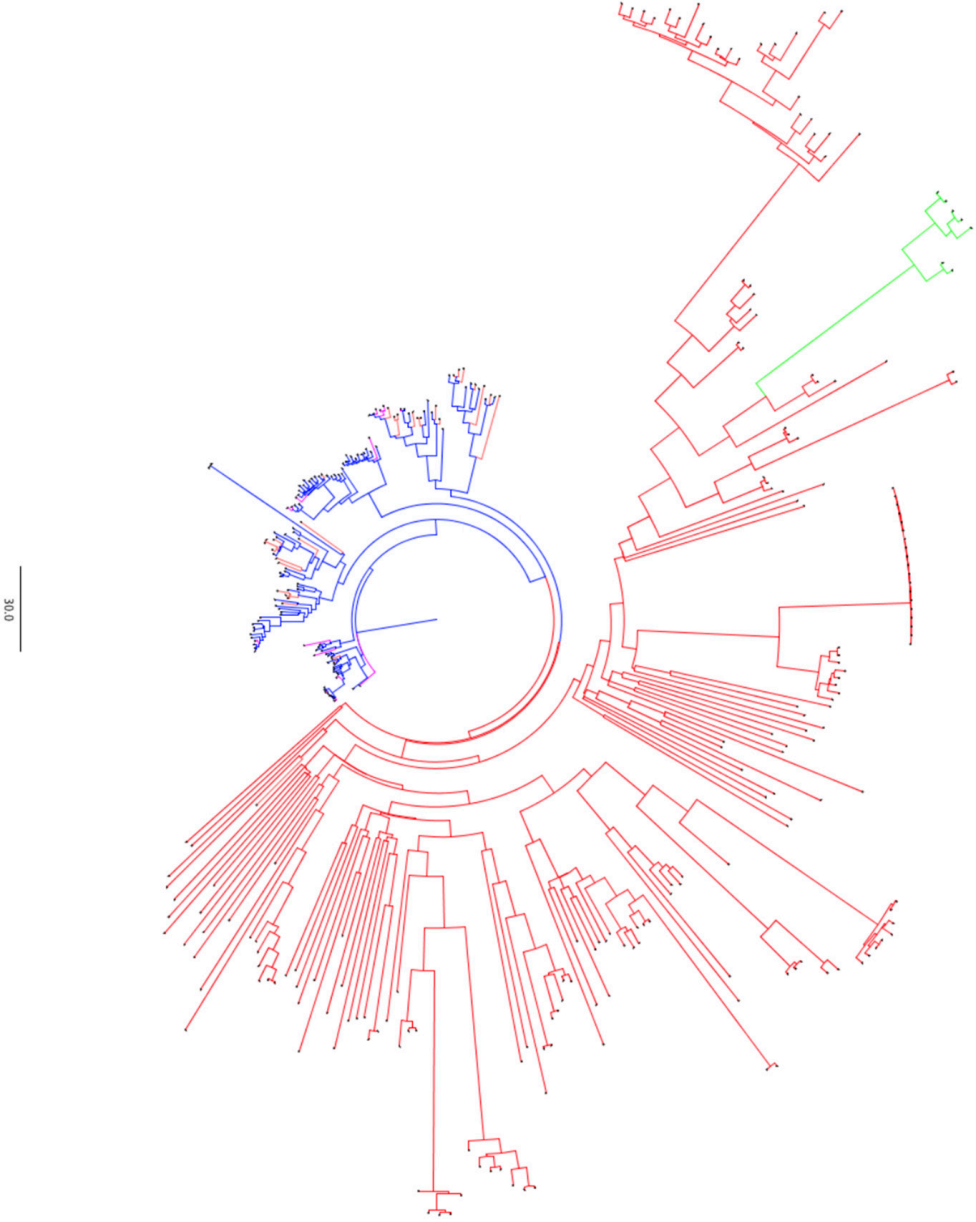
Maximum likelihood phylogenetic tree generated using IQ-TREE from the sequence alignment of all the predicted RGAs characterized in the ‘CATD’ genome assembly. JTT amino acid substitution model (Jones *et al.* 1992) with empirical codon frequencies (+F) and FreeRate (+R9) rate heterogeneity across sites (Yang 1995; Soubrier *et al.* 2012) was used to generate the tree, validated with 1000 replicates of ultrafast bootstrapping (Hoang *et al.* 2017) and SH-aLRT (Guindon *et al.* 2010) tests. The branches colored as red are for TM-CC, blue for NBS-containing and green for TX and TN resistance gene analogs.

The majority of the predicted RGAs could be classified into two major clades and two major RGA families, *i.e.*, NBS-containing and TM-CC (Figure 4). This corresponds to the automatic RGA classification, of which the major classes within a major RGA gene family could be arranged into a clade. TM-CC clade is composed of two sub-clades: one is more similarly related to the NBS-containing domain clade, while the other is more related to the TX- and TN-comprising clade. Although most of the NBS-domain containing clade could be arranged into a single clade, the specific RGA classes within the NBS clade are mixed in separate sub-clades. The maximum likelihood tree constructed based on the characterized coconut RGAs will provide basic knowledge on the evolution of resistance genes during the course of crop’s adaptation to disease pressure caused by a wide-array of pathogens. As a baseline mechanism of coconut host-response, this genome-wide RGA mapping will provide the development framework to target candidate genes for efficient pyramiding in disease resistance breeding. Example targeting RGAs of a clade with conserved domain/motif specific against a pathogen, or incorporating all RGAs to represent each clade and sub-classes for a broad-based host resistance. However, it is critical to perform further functional analysis (*e.g.*, differential gene expression, knock-out assays) and marker-phenotype association studies (*e.g.*, QTL mapping, fine-mapping) for a specific disease to validate the utility of any of these RGAs in actual plant varietal improvement programs.

### Drought-response genes

Although coconut is classified as relatively a drought tolerant crop, moisture scarcity is among its major constraints in worldwide coconut production (Prasada Rao 1986). Furthermore, drought is also reported to affect the growth and physiology of the crop (Repellin *et al.* 1997; Bai and Rajagopal 2000; Prado *et al.* 2001; Gomes *et al.* 2009; Passos *et al.* 2009; Gomes and Prado 2010). Similar to salinity and waterlogging stress, drought is controlled by several quantitative trait loci (QTL) which makes drought-resilient coconut varietal improvement difficult through traditional breeding methods. With the availability of genome-wide gene models controlling drought traits, precise breeding methods through new breeding techniques (NBTs) can be explored for varietal improvement for drought resilient varieties (Fita *et al.* 2015; Gao 2018).

A total of 199 drought-response genes were downloaded from DroughtDB (Alter *et al.* 2015), a public database containing all drought-related genes that have been characterized in several crops. Through BLAST (E-value: 0.0), the homology sequence alignment of these genes was investigated against the genome-wide gene models of the current coconut genome assembly. Only 62 (31.1%) of the drought-response genes from DroughtDB were found to have at least one (1) homolog in the dwarf coconut genome. In total, there are 213 drought-related gene homologs in coconut (Data S4). The ABCG40 gene (AT1G15520) has the highest number of homologs - thus far 15 coconut gene homologs. This gene functions as ATP-binding cassette (ABC) transporters and abscisic acid (ABA) importers, and therefore can mediate cellular uptake of phytohormone abscisic acid (Kang *et al.* 2010).

The distribution of the biological function of all the identified candidate drought-response gene homologs was generated by analyzing the GO terms of each candidate gene model (Figure S8). The intracellular signal transduction activity has the most number of sequences, followed by oxidation-reduction processes and abscisic acid-activated signaling pathway.

### Genome-wide DNA markers for coconut

About 39,002 SSR loci were detected in the coconut ‘CATD’ genome using the GMATA software package (Wang and Wang 2016) with parameters as described in the Materials and Methods section of this paper. GMATA package is a tool for the identification of SSR loci, statistical classification, and SSR marker development. Di-repeats (or 2-mer) were observed as the most commonly found repeat motif (Figure S9). Among these, AT/TA (69.9%) is the top paired SSR repeat motif followed by CT/AG (21.6%) and TG/CA (4%) (Figure S10). Overall, the frequency of SSR loci in dwarf coconut as represented by ‘CATD’ in this study, can be characterized to be 25.11 per million base pair of the whole genome. A total of 22,031 unique SSR markers were designed or 61% of the total SSR loci mapped in the ‘CATD’ genome. To increase the specificity and robustness of SSRs for various applications, the AT/TA repeat motifs were excluded, coming up with a final set of 7,139 coconut SSR markers (Data S5).

Aside from neutral genome-wide SSR markers, 13 gene-linked SSRs were also designed from the whole genome assembly of ‘CATD’. Using Primer3 software in the GMATA package, SSR markers tagging the genes/loci of interest were generated. Genes tagged included four (4) representative genes associated to drought response, five (5) for insect and pest resistance, and four (4) genes for oil biosynthesis (Table S5). As a result, 39 (drought response genes), 157 (insect and pest resistance), and 25 (oil biosynthesis) SSR markers were designed with definite physical distance to each target gene. The information of physical linkage of markers to target genes is invaluable for downstream applications such as fine mapping and trait/phenotype association studies toward genetic improvement to develop stress-resilient and oil outstanding coconut varieties.

To aid in the development of SNP genotyping arrays and for other high-throughput genotyping technologies such as genotyping-by-sequencing (GBS), sequence variations such as SNPs and InDels between the reference Dwarf coconut (current ‘Catigan Green Dwarf’ or ‘CATD’) genome and the reference Tall coconut genome (Hainan Tall) were characterized. The raw reads (SRR5273820, SRR5273822) from the whole genome sequencing of Hainan Tall (Xiao *et al.* 2017) were quality trimmed generating a total of 234 million high-quality paired-end reads. Around 21.7 Gbp high-quality PE reads of Hainan Tall were mapped to the ‘CATD’ genome represented by a sequence alignment map (SAM) file. The repeat-masked ‘CATD’ genome was used in the mapping step to determine unique consensus sequences and to minimize misalignment of reads especially involving repeat sequences, which can be problematic in downstream variant discovery (Johnston *et al.* 2017). Upon processing and generating a BAM file, the mapping analysis returned a coverage depth mean value of 38.82%; that is the alignment coverage of Hainan Tall genome-PE reads with that of the repeat-masked genome of ‘CATD’. Prior to base quality score recalibration, an initial raw set of 1,441,887 variants were detected between the mapped genomes using the GATK Best Practices workflow (Van Der Auwera *et al.* 2013; see specific section in the Materials and Methods). Recalibrating the base quality scores of the mapped reads resulted to a reduced set of 58,503 variants and all located in the non-repeat regions of the coconut genome. The summary, characteristics and distribution of these variants within specific regions of the coconut genome are presented in Table 2.

**Table 2:** Summary, characteristics and distribution of sequence variations between ‘Hainan Tall’ and ‘Catigan Green Dwarf’ (CATD) genomes. Location of variants is based on ‘CATD’ sequence assembly as the reference in this genome mapping analysis

	Genome Region		
Variants	Non-repeat region	Genic region	Exonic region
	(intergenic, gene)	(Intron + Exon)	
Number of SNPs	57,872	21,066	5,552
Number of Transversions	40,233	7,192	1,664
Number of Transitions	17,639	13,875	3,888
Ts/Tv ratio	2.2809	1.93	2.34
Number of InDels (1-6 bp)	631	143	48
Single-base InDels	392	70	17
di-nucleotide InDels	128	32	12
>3-bp InDels	111	41	19
Total Number of Variants	58,503	21,209	5,600

**Figure 5:**
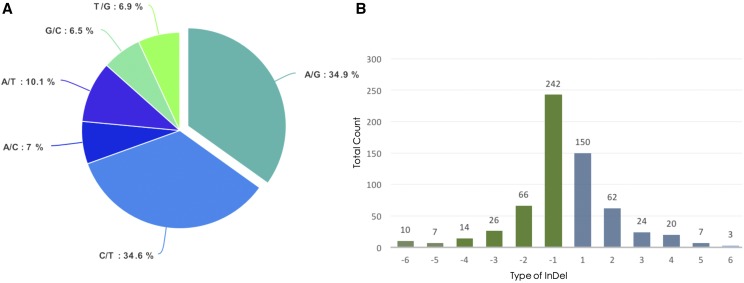
Occurrence of sequence variations in the non-repeat region of coconut based on map alignment of ‘HAT’ WGS reads to the assembled ‘CATD’ genome. (a) Distribution of the type of coconut SNPs (transversions and transitions) detected; (b) frequency of occurrence of each SNP and bp length of InDels identified in coconut. Negative values signify deletion while positive values are insertions relative to the sequence of the assembled ‘CATD’ genome.

Around 98.9% of the variants detected is composed of SNPs at non-repeat regions between ‘Catigan Green Dwarf’ and ‘Hainan Tall’ genomes (Table 2). Among these SNPs, 69.52% are characterized as single-base transitions (A/G, C/T), while the rest are classified as transversion substitutions (A/C, A/T, G/C, T/G) (Figure 5a). A/G transition is the most frequent base substitution - 20,221 loci or 34.56% of the total sequence variants detected. The frequency of SNP mapped can be translated to one (1) SNP every 7,881 bp sequence in the non-repeat region of the coconut genome. Meanwhile, a total of 631 InDels ranging 1-6 bp were identified among the set of variants (Figure 5b), of which most are characterized as single-base InDels (62.12%) followed by di-nucleotide InDels (20.29%). Considering the totality of the variants detected, the nucleotide diversity of the non-repeat region (456.1 Mbp) between the ‘Dwarf’ and ‘Tall’ coconut genomes could be estimated to be 128 base difference per Mbp.

With the information of genome location of genes for several economically important traits, targeted SSR marker development and SNP-based genotyping systems for each trait is now possible. Meanwhile. the genome-wide SSR marker would be an invaluable genetic resource for a wide-array of applications in coconut such as assessment of genetic diversity (Oyoo *et al.* 2016), fingerprinting (Preethi *et al.* 2016; Kamaral *et al.* 2017), ecological-genetic studies (Geethanjali, *et al.* 2018), gene flow characterization (Larekeng *et al.* 2015) and fine- mapping genetic studies (Riedel *et al.* 2009). On the other hand, high-throughput and SNP-based marker assays such as microarray and genotyping-by-sequencing can now be applied in coconut breeding. The SNP information identified through the mapping of the whole genome sequence (WGS) reads of recently published ‘Hainan Tall’ genome to the assembled whole genome of ‘Catigan Green Dwarf’ coconut will provide a platform to accelerate the current status of the international coconut genetics and genomics programs, in particular toward the development of stress-resilient, outstanding and special types coconut varieties.
